# One Assay to Rule Them All: Development of a Global Environmental DNA Tool to Support Range‐Wide Surveys of an Imperiled Sawfish and Its Application in the Eastern Tropical Pacific

**DOI:** 10.1002/ece3.73733

**Published:** 2026-06-02

**Authors:** Juan C. Cubillos‐M, Annmarie Fearing, Demian D. Chapman, Peter M. Kyne, P. Joana Dias, Cindy Gonzalez, Ryan N. Lehman, Yaliana Chichaco, Bryan L. Huerta‐Beltrán, Kayla L. McCulloch, Sarah M. Toepfer, John K. Carlson, Nicole M. Phillips

**Affiliations:** ^1^ Thünen Institute of Sea Fisheries Bremerhaven Germany; ^2^ Ecological Genomics Group, Institute of Biology and Environmental Sciences University of Oldenburg Oldenburg Germany; ^3^ School of Biological, Environmental, and Earth Sciences The University of Southern Mississippi Hattiesburg Mississippi USA; ^4^ Sharks and Rays Conservation Research Program Mote Marine Laboratory & Aquarium Sarasota Florida USA; ^5^ Research Institute for the Environment and Livelihoods Charles Darwin University Darwin Northern Territory Australia; ^6^ Aurora Biodiversity LLC Hattiesburg Mississippi USA; ^7^ Predator Ecology and Conservation Lab, Biological Sciences Department Florida International University North Miami Florida USA; ^8^ Smithsonian Tropical Research Institute Smithsonian Institution Panama City Panama; ^9^ Department and Marine and Environmental Sciences Savannah State University Savannah Georgia USA; ^10^ National Marine Fisheries Service Southeast Fisheries Science Center Panama City Florida USA

**Keywords:** Colombia, digital PCR, Habitat Suitability Index, Largetooth Sawfish, Panama, rhino ray

## Abstract

Environmental DNA (eDNA) has emerged as a powerful tool to support the conservation of threatened species. The sawfishes are one of the most imperiled vertebrate groups, with all five species classified as Critically Endangered on the IUCN Red List of Threatened Species. Owing to their toothy rostra, sawfishes are easily entangled in a wide range of fishing gear, with mortalities in fisheries and loss of habitat driving global declines. The Largetooth Sawfish, *Pristis pristis* , historically had a circumtropical range, but has experienced severe declines in geographic range and abundance. Viable populations are thought to be confined to northern Australia, but a handful of other nations may harbor remnant populations. To identify promising locations where populations may persist, a novel species‐specific Droplet Digital PCR (ddPCR) assay was designed capable of detecting a single copy of DNA (Limit of Detection 0.07 copies/μL), with in silico and in vitro validation experiments indicating functionality across the species' historical range. Water samples were then collected in 2022 at 99 sites in the Eastern Tropical Pacific from the Gulf of Chiriquí (*N* = 48) and Darién (*N* = 18) regions of Panama, and the Chocó region (*N* = 33) of Colombia, but *P. pristis* DNA was not detected at any sampling sites. Habitat Suitability Index modeling revealed that sampling sites were located within, or in proximity to, basins with highly suitable habitats for sawfish. Detecting rare species in remote data‐poor regions presents major challenges, and additional spatially and temporally comprehensive eDNA surveys using this novel ddPCR eDNA assay are recommended. These surveys should focus on locations where the presence of *P. pristis* remains uncertain to identify priority locations for global conservation efforts.

## Graphical abstract

To identify promising locations where Largetooth Sawfish, *Pristis pristis* , may persist, a novel species‐specific Droplet Digital PCR (ddPCR) assay was designed capable of detecting a single copy of DNA, with in silico and in vitro validation experiments indicating functionality across the species' historical range. Water samples were then collected in 2022 at 99 sites in the Eastern Tropical Pacific from the Gulf of Chiriquí and Darién regions of Panama, and the Chocó region of Colombia, but *P. pristis* DNA was not detected at any sampling sites. Detecting rare species in remote data‐poor regions presents major challenges, and additional spatially and temporally comprehensive eDNA surveys using this novel ddPCR assay are recommended.

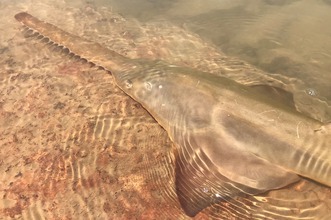

## 1 Introduction

Environmental DNA (eDNA) is a powerful tool to support the conservation of highly threatened species. All organisms shed DNA into their environment, and in aquatic systems, this eDNA can be recovered by filtering water samples and species identified via genetic analyses (Jerde et al. 2011). eDNA techniques are being used to assess presence, distribution, habitat use, biomass/abundance, and to support monitoring efforts for threatened aquatic species (Jerde et al. 2011; Capo et al. 2021), often outperforming traditional survey methods including capture‐based and visual search surveys (Fediajevaite et al. 2021). Species‐specific eDNA approaches use custom genetic assays that are designed to only “detect” DNA for the species of interest, but not from closely related co‐occurring species (“exclusion species”), allowing for high specificity of a target species (Jerde et al. 2011; Wilcox et al. 2013). Coupling species‐specific eDNA approaches with advanced digital PCR technology, such as Droplet Digital PCR (ddPCR), allows for the detection of a single copy of DNA, increasing detectability when target DNA is present in a reaction (Wood et al. 2019; Claver et al. 2026). This unparalleled sensitivity makes eDNA an immensely valuable tool when conducting field surveys for highly imperiled species, especially in dynamic and expansive estuarine and marine ecosystems where traditional sampling techniques may not be feasible.

eDNA approaches have the potential to fill data gaps for threatened aquatic species to support more strategic conservation actions. Sharks and rays (class Chondrichthyes; sub‐class Elasmobranchii) are among the most threatened aquatic taxa, with ~33% of species threatened with extinction (IUCN 2025). Rays (superorder Batoidea) face a greater overall risk of extinction than sharks, with the sawfishes (Pristidae), wedgefishes (Rhinidae), and giant guitarfishes (Glaucostegidae) (three of five families within the order Rhinopristiformes or “rhino rays”) being among the most imperiled vertebrate groups on the planet (Kyne et al. 2024). Sawfishes are large benthic rays with a long toothy rostrum that inhabit shallow coastal and, in some cases, riverine waters. All five species are classified as Critically Endangered on the IUCN Red List of Threatened Species, threatened primarily due to their low intrinsic rates of population increase, high catchability in fisheries, high market value for fins and rostra, and loss of habitat (IUCN 2025). Over the past century, the geographic distributions of all sawfishes have been greatly diminished. Historically, sawfishes were distributed in the coastal waters of 90 countries and territories but are presumed extinct in at least 46, with 18 countries having lost at least one species altogether (Yan et al. 2021). The presence of sawfish remains uncertain in 42 countries where they historically occurred, largely due to difficulties in detecting rare species in remote areas using traditional survey methods (Yan et al. 2021).

The Largetooth Sawfish, *Pristis pristis* , historically had a circumtropical range, but has undergone severe global declines (Espinoza et al. 2022). *Pristis pristis* is unique among the sawfishes for its use of freshwater rivers as juveniles; however, this life history strategy makes it susceptible to a multitude of threats across both marine and freshwater habitats (Grant et al. 2019). Viable populations are now likely restricted to northern Australia, although *P. pristis* persists at lower abundances in a handful of other regions (Espinoza et al. 2022). In an analysis using dynamic geography theory, eight nations were identified as priority locations for targeted sawfish surveys based on a very low probability of extinction, but where presence was uncertain (Yan et al. 2021). Of these identified nations, numerous reports of *P. pristis* have emerged in the last decade from the Pacific coasts of Panama and Colombia (López‐Angarita, Cubillos‐M, et al. 2021). Using kernel density estimation of sawfish encounter locations across the Pacific coasts of Panama and Colombia, the Darién (Panama) and Chocó (Colombia) regions were recently identified as promising sawfish ‘bright spots’ (López‐Angarita, Cubillos‐M, et al. 2021). Additional locations with numerous reports of sawfish in this region from 2010–2015 included Chiriquí, Coclé, and Veraguas in Panama (López‐Angarita, Cubillos‐M, et al. 2021). These regions are all broadly characterized by their remoteness and high mangrove forest cover, which *P. pristis* relies heavily on as nursery habitat (Whitty et al. 2017; López‐Angarita, Cubillos‐M, et al. 2021).

Given the remoteness of tropical regions potentially harboring remnant *P. pristis* populations, resolving the uncertainties of its geographic range requires the use of highly sensitive, technologically advanced, and globally scalable eDNA surveys. Three *P. pristis* eDNA assays were previously designed for specific regional populations, two for use in Australia and a third for use in Brazil (Cooper et al. 2021; Rodrigues et al. 2025), limiting the broader utility of the assays outside of these regions. Further, these assays used different types of PCR platforms, hindering comparisons among surveys, owing to differences in assay sensitivities (Simpfendorfer et al. 2016; Cooper et al. 2021; Rodrigues et al. 2025).

Here, we developed a ddPCR eDNA assay with functionality across the historic range of *P. pristis* to support range‐wide global eDNA surveys. eDNA surveys were then conducted in priority locations in the Eastern Tropical Pacific where presence is uncertain, the Darién and Gulf of Chiriquí regions of Panama, and the Chocó region of Colombia. To evaluate habitat suitability of the locations selected for eDNA sampling and prioritize locations for future survey efforts, a spatial composite Habitat Suitability Index (HSI) was developed a posteriori for freshwater and estuarine habitats of Pacific Panama and Colombia.

## 2 Methods

### 2.1 Field and Laboratory Controls

All field and laboratory methods scrupulously adhered to rigorous protocols and controls to reduce the likelihood of contamination of exogenous DNA and cross‐contamination of samples (i.e., Lehman et al. 2022; Dias et al. 2025). Briefly, during fieldwork, the boat was cleaned with 10% bleach each day before sample collection, and between sites when any seawater got into the designated clean eDNA workspace on the boat. eDNA field personnel remained in a designated clean eDNA workspace and did not handle any non‐eDNA equipment (e.g., ropes, anchors). All supplies for water collection and filtration were cleaned prior to each use with 10% bleach, followed by UV sterilization for 20 min, and new supplies were used to collect and filter each water sample. The benthic sampler used for collecting water samples was cleaned between each use as described in Lehman et al. (2022), and when samples were collected by hand, all materials, including clothing, were cleaned with 10% bleach between collection sites. Water filtration occurred in a designated workspace in the field, isolated from both used and clean eDNA materials. The filtration workstations and equipment were also cleaned with 10% bleach between each sample.

Laboratory processing used uni‐directional workflows, with eDNA extractions and ddPCRs spatially (e.g., separate laboratory spaces and equipment) and temporally (e.g., different days) isolated, and all reagents were stored in refrigerators and freezers without DNA present. Project‐specific pipettes with aerosol barrier tips were used for the duration of the study, and separate sets of pipettes were used for each stage in sample processing to reduce carry‐over. Assay development using genomic DNA (gDNA) and synthetic DNA occurred in laboratories separate from those used for processing eDNA samples, also following unidirectional workflows.

Negative controls were incorporated into each stage and day of sample processing (e.g., fieldwork, DNA extraction, PCR), and all were analyzed with the optimized ddPCR eDNA assay, in replicates of five, to test for the possibility of contamination. Negative field controls consisted of bringing 3‐L of distilled bottled water (i.e., the cleanest available) into the field each day in clean 1‐L clean Nalgene bottles, which were treated identically to field samples; for example, stored on ice in the same cooler, poured through the benthic samples and/or opened for the same amount of time as field samples. Negative controls for DNA extractions included all reagents, but did not include filters, and negative controls for ddPCR included all reagents, but used PCR‐grade water rather than DNA template.

### 2.2 eDNA Assay Design and Validation

A novel species‐specific ddPCR eDNA assay was designed to amplify DNA from *P. pristis* , but not from co‐occurring exclusion species. To design this assay, mitochondrial DNA (mtDNA) 12S ribosomal RNA (rRNA) sequences for *P. pristis* and the three other *Pristis* spp. were aligned in CodonCode v. 6.0.2 (CodonCode Corporation, Dedham, MA, USA) (Table 1). Forward (5′‐ACATCGCTAAAACCATCTACC‐3′) and reverse (5′‐GGTTGATGGCAAGAAGTGGT‐3′) primers and an internal PrimeTime double‐quenched ZEN/IOWA Black FQ probe (5′‐CGGTGCCTCAGACCCACCT‐3′) labeled with 6‐FAM were designed to amplify a 113‐base pair (bp) fragment of the target gene in only *P. pristis*. This was achieved by designing primers and probes in conserved regions within *P. pristis* while including a minimum of 9 total bp differences in other *Pristis* spp. (Table 2).

**TABLE 1:** List of target species, Largetooth Sawfish, *Pristis pristis* , and three exclusion species representing all species of the genus *Pristis* used to design species‐specific primers and an internal probe to amplify a 113‐base pair fragment of the mitochondrial 12S rRNA gene in only *P. pristis*.

Species	GenBank accession no.	Collection location
*Pristis pristis*	NC_039438	Daly River, Northern Territory, Australia
*Pristis clavata*	KF381507	South Alligator River, Northern Territory, Australia
*Pristis pectinata*	NC_027182	Turner River, Florida, United States
*Pristis zijsron*	MH005927	Cape Keraudren, Western Australia, Australia

**TABLE 2:** Rhino ray exclusion species used for in silico assay specificity validation. Numbers are the base pair differences between the species‐specific primers and probe designed for Largetooth Sawfish, *Pristis pristis*, and each exclusion species.

Species	GenBank accession no.	Forward primer	Reverse primer	Probe	Total no. of base pair differences
Pristidae					
*Anoxypristis cuspidata*	NC_026307	6	2	1	9
*Pristis clavata*	KF381507	5	2	2	9
*Pristis pectinata*	NC_027182	4	4	6	14
*Pristis zijsron*	MH0052927	7	2	2	11
Rhinidae					
*Rhina ancylostoma*	KU721837	7	3	2	12
*Rhynchobatus australiae*	NC_030254	7	1	2	10
*Rhynchobatus djiddensis*	NC_066688	7	1	1	9
*Rhynchobatus laevis*	NC_047241	7	0	3	10
Glaucostegidae					
*Glaucostegus cemiculus*	PQ773615	5	0	2	7
*Glaucostegus granulatus*	MN783017	5	0	2	7
*Glaucostegus typus*	MN795525	5	1	2	8
Rhinobatidae					
*Acroteriobatus annulatus*	NC_068897	7	3	1	11
*Pseudobatos horkelli*	PQ014263	7	1	2	10
*Rhinobatos annandalei*	PV430499	8	2	1	11
*Rhinobatos sainsburyi*	ON889554	7	3	1	11

*Note:* This covers all species within the family Pristidae (sawfishes: *Anoxypristis*, *Pristis*), two of three genera of Rhinidae (wedgefishes: *Rhynchobatus*, *Rhina*), the single genus of Glaucostegidae (giant guitarfish: *Glaucostegus*), and all three genera of Rhinobatidae (guitarfish: *Acroteriobatus*, *Rhinobatos*, *Pseudobatos*). Mitochondrial 12S rRNA was not available for the remaining wedgefish genus (*Rhynchorhina*) or the remaining rhino ray family Trygonorrhinidae (banjo rays).

To ensure the genetic assay amplified the mtDNA 12S rRNA gene, quantitative real‐time PCR (qPCR) was conducted using tissue‐derived DNA from two *P. pristis* individuals, one from each of the Indo‐West Pacific and Eastern Pacific. Reaction mixtures contained ~25 ng of DNA, 1X Applied Biosystems Power SYBR Green PCR Master Mix, 200 nanomolar (nM) of each primer, adjusted to 22 μL using PCR‐grade water. Cycling conditions consisted of enzyme activation at 95°C for 10 min, followed by 44 cycles of: 95°C for 15 s and 55°C for 30 s, followed by enzyme deactivation at 60°C for 1 min, using a ramp rate of 1°C/s. The amplicon from the Eastern Pacific *P. pristis* was cleaned using a QIAGEN QIAquick PCR Purification Kit using the manufacturer's protocol, with the exception that all centrifugation steps were conducted at 10,000 rpm and/or 12,000 rpm for 2 min. The amplicon was sequenced in the forward direction using a BigDye Terminator v3.1 Cycle Sequencing Kit (Applied Biosystems, Foster City, CA, USA) on an Applied Biosystems 3730XL DNA Analyzer. The identity of the sequence was verified as the target locus via the NCBI Blastn search function and was 100% similar to *P. pristis* GenBank accession no. NC_039438 (Kyne et al. 2018).

PCR reaction and cycling conditions were optimized for the Bio‐Rad QX200 AutoDG Droplet Digital PCR System (Instrument no. 773BR1456, 771BR2544) to produce positive droplets with high relative fluorescence units (RFUs). Optimized ddPCR reaction mixtures contained 1.1 μL of extracted DNA, 1X Bio‐Rad ddPCR supermix for probes (no deoxyuridine triphosphate; dUTP), 900 nM of each primer, and 250 nM of probe, adjusted to 22 μL using PCR‐grade water, as per the manufacturer's protocol for automated droplet generation (Bio‐Rad Laboratories 2014). Using an automated droplet generator, 20 μL of each ddPCR reaction mixture was combined with ~70 μL of automated droplet generation oil to create ~20,000 nanoliter‐sized droplets prior to PCR cycling (Bio‐Rad Laboratories 2014). Optimal ddPCR cycling conditions were enzyme activation at 95°C for 10 min, followed by 40 cycles of: 94°C for 30 s and 60°C for 2 min, with a final enzyme deactivation step at 98°C for 10 min, using a ramp rate of 1°C/s.

The ddPCR eDNA assay was validated in vitro using *P. pristis* DNA extracted from dried rostra specimens collected across their historical global range (see Phillips et al. 2009). These specimens were identified as *P. pristis* using morphological characteristics, such as rostra shape and tooth count and spacing (see Faria et al. 2013; Whitty et al. 2014), and genetic analysis of the mtDNA control region. The assay was validated using a minimum of three individuals from each of the four regional sub‐populations (Faria et al. 2013): the Indo‐West Pacific (Bangladesh, Papua New Guinea, Australia; *N* = 3), Eastern Pacific (Panama, Ecuador, Peru; *N* = 4), Western Atlantic (United States, Nicaragua, Brazil; *N* = 4), and Eastern Atlantic (The Gambia; N = 3).

The ddPCR eDNA assay was tested for species‐specificity in silico and in vitro. The assay was validated in silico using NCBI Blastn (Altschul et al. 1990) to evaluate range‐wide species‐specificity (Table 2). The optimized ddPCR reaction and cycling conditions were then cross‐tested in vitro with 0.20 ng of tissue‐derived DNA from eight representative exclusion species, in triplicate reactions. This in vitro assay validation included testing with all four other sawfish species found globally and four guitarfishes (*Pseudobatos* spp.) from across the Eastern Pacific and Western Atlantic (Table 3).

**TABLE 3:** Exclusion species that the Droplet Digital PCR eDNA assay was cross‐tested with in vitro to ensure the assay was species‐specific for Largetooth Sawfish, *Pristis pristis* , in the Eastern Pacific and Western Atlantic.

Species	Ocean basin	Collection location
*Anoxypristis cuspidata*	Indian	India
*Pristis clavata*	Indian	Australia
*Pristis pectinata*	Atlantic	West Africa
*Pseudobatos lentiginosus*	Atlantic	Gulf of Mexico, Florida, USA
*Pseudobatos buthi*	Pacific	Gulf of California, Mexico
*Pseudobatos glaucostigma*	Pacific	Gulf of California, Mexico
*Pseudobatos productus*	Pacific	California, USA

### 2.3 eDNA Surveys

**FIGURE 1:**
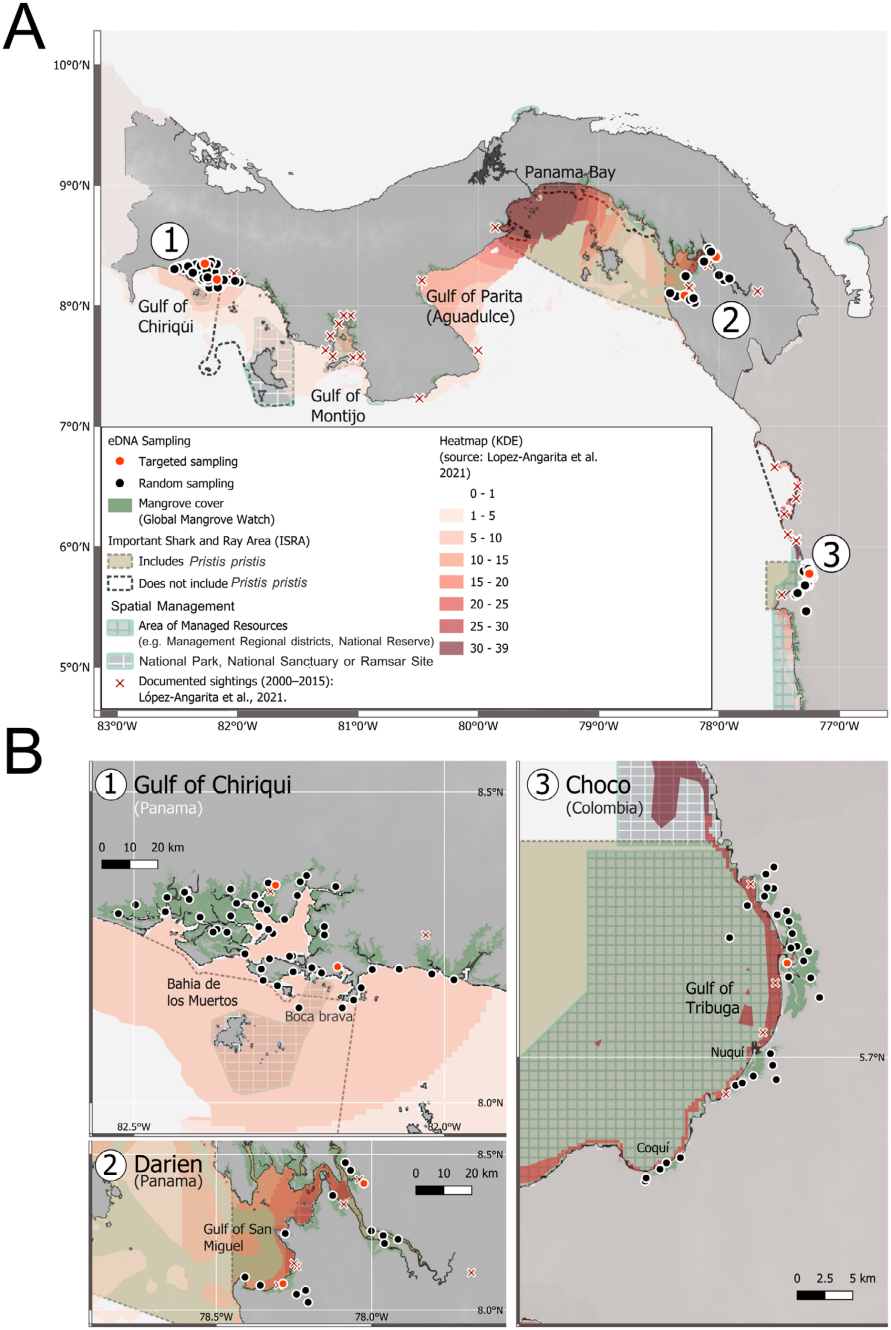
Sampling sites of environmental DNA (eDNA) surveys for Largetooth Sawfish, *Pristis pristis* , in the Eastern Tropical Pacific. (A) The three sampling regions in Panama (1, Gulf of Chiriquí; 2, Darién) and Colombia (3, Chocó) in bright spots for sawfish (modified from Kernel Density Estimate [KDE], López‐Angarita, Cubillos‐M, et al. 2021) alongside the records of sightings since 2000, indicated by red X's. (B) Sampling sites (circles), where red indicates a directed site and black indicates a randomly generated site in: (1) Gulf of Chiriquí and (2) Darién regions in Panama, and (3) Chocó region in Colombia. Mangrove cover is shown in green, spatial management in gridded polygons, and Important Shark and Ray Areas (ISRAs) in dashed lines, with ISRAs where *P. pristis* is a Qualifying Species highlighted.

Water samples were collected from 99 sites in the Eastern Tropical Pacific in 2022, consisting of 66 in Panama in June and 33 in Colombia in July. The Gulf of Chiriquí (*N* = 48; within and adjacent to Bahía de los Muertos) and the Darién (*N* = 18) regions were surveyed in Panama, while in Colombia, all samples were from the northern Chocó region. Within each region, two sites with historic sawfish reports (see López‐Angarita, Cubillos‐M, et al. 2021) were directly targeted for sampling (Figure 1). In Panama, these sites were in Boca Chica and Rio Chiriquí in the Gulf of Chiriquí and in the Tuira and Sambu rivers in the Darién. In Colombia, these sites were in the Gulf of Tribugá and Coquí. All remaining sites in each region were selected using a random sampling approach described in Poulakis et al. (2011) and Lehman et al. (2022) with a maximum sampling depth of five meters (Figure 1).

At each site, a 3‐L benthic water sample was collected either by hand or via a custom‐made benthic sampler (see Lehman et al. 2022). Samples were stored in clean 1‐L polyethylene Nalgene bottles in a cooler with ice until filtration, which was completed within 12 h of collection. After water sample collection, water temperature and salinity data were collected at the surface and the benthos at each site, as well as turbidity, measured using a secchi disk. Salinity and turbidity data were only able to be collected in the Gulf of Chiriquí due to equipment limitations in the Darién and Chocó regions. Water samples were filtered using Grover‐Go (Grover Scientific, Rosslea, Australia) portable vacuum pumps and self‐preserving filter housing (Smith‐Root, Vancouver, Washington, USA) with 5 μm 47‐mm nitrocellulose filters (Whatman, Maidstone, United Kingdom), then stored at 20°C until they could be archived at −80°C (within 6 months of collection). Typically, two filters were needed for each 3‐L water sample, although the number of filters ranged from one to four depending on sample turbidity. A 3‐L negative control water sample consisting of bottled water was included each day of eDNA surveys as previously described. eDNA was extracted from half of each filter, and when there was more than one filter for a sample, the halves of all filters were pooled into a single DNA extraction. Total eDNA was recovered from samples using the QIAGEN DNeasy Blood & Tissue Kit (Qiagen, Hilden, Germany) following the Goldberg et al. (2011) protocol incorporating QIAshredder spin columns and eluted with 50 μL of elution buffer. eDNA extracts were quantified using NanoDrop technology (ThermoFisher Scientific, Waltham, Massachusetts, USA), and their qualities assessed via visualization on a 2% agarose gel. eDNA extracts were screened for *P. pristis* DNA using the optimized ddPCR reaction mixtures and cycling conditions in replicates of five, screening ~10% of each DNA extract. Despite ddPCR being less prone to inhibitors (Dingle et al. 2013; Rački et al. 2014), 26 samples (11 from the Darién and 15 from Chocó) were re‐run with 0.66 μL Bovine Serum Albumin (BSA, 20 mg/mL) in replicates of five to evaluate the potential of false negatives due to PCR inhibition.

### 2.4 Data Analysis

All eDNA samples and negative controls were analyzed using Rare Event Detection (RED) in the BioRad QuantaSoft software. Samples were defined as positive detections of *P. pristis* DNA when at least one of the five replicates met three analysis criteria: (1) droplets were above the manual threshold (MT) of the assay, (2) droplets fell within the known positive droplet range for the assay, defined during in vitro assay validation, and (3) the concentration of target DNA was greater than or equal to the Limit of Detection (LoD) of a single positive droplet detection (see Dias et al. 2025). This approach to data analysis guarded against false positive detections, such as incorrectly calling artifact droplets as positive detections. To meticulously consider the possibility of contamination, for negative controls to be deemed free from contamination, none of the replicates could meet any of the three criteria for positive detections (e.g., no droplet above the MT).

To distinguish target DNA from artifact droplets, the MT for the assay was determined through the analysis of 48 ddPCR no template control (NTC) reactions containing no target DNA. After the MT was defined, the LoD was determined via screening a series of 10× dilutions from starting concentrations of 0.2 ng/μL synthetic gBlock Gene Fragments (Integrated DNA Technologies, Coralville, Iowa, USA) for the target locus, in triplicate reactions. Target DNA was detected in the replicates of the 2.0 × 10^−9^ ng/μL dilution, but in none of the replicates of the 2.0 × 10^−10^ ng/μL dilution. To refine the LoD further and achieve “one‐droplet” detections (Dias et al. 2025), a threefold series of dilutions were then analyzed from the 2.0 × 10^−9^ ng/μL dilution. The LoD was then determined by averaging the number of copies of target DNA/μL in one‐droplet detections and applying the lower standard error as the relaxed detection threshold (see Baker et al. 2018; Lehman et al. 2020).

### 2.5 Habitat Suitability Index Model (HSI)

Sampling effort across the Pacific drainage basins of Panama and Colombia was evaluated using a spatial HSI model. Given the different spatial resolutions of HSI modeling and eDNA surveys, the HSI model was developed at the drainage‐basin scale to assess regional habitat suitability and complement finer‐scale eDNA sampling. Specifically, the proximity of eDNA sampling sites to highly suitable *P. pristis* habitat and previously described ‘bright spots’ based on historical sightings (López‐Angarita, Cubillos‐M, et al. 2021) was assessed. Four geospatial layers were compiled, representing environmental variables likely to influence *P. pristis* habitat use: (1) flow accumulation, used as a proxy for river size and discharge; derived from watershed analysis using Digital Elevation Models (DEM) (see Lehner et al. 2008, accessed via https://www.hydrosheds.org/products/hydrosheds), (2) proximity to mangrove habitats, calculated from mangrove cover data based on the Global Mangrove Watch (GMV, https://www.globalmangrovewatch.org), (3) shallow shelf area, derived from GEBCO bathymetry grid (GEBCO, https://www.gebco.net) and constrained to a depth of 25 m to reflect critical nearshore habitat preference (Yan et al. 2021), and (4) proximity to historical sighting locations, represented by the Kernel Density Estimate (KDE) of recorded occurrences (López‐Angarita, Cubillos‐M, et al. 2021). All layers were normalized to a scale of 0–1. The selected remote sensing variables do not comprehensively capture all dimensions of habitat suitability for *P. pristis* , but represent the best available proxies for data‐limited and remote regions (e.g., Yan et al. 2021).

The four normalized layers were combined using a weighted geometric mean (WGM) method to calculate a composite HSI (Zajac et al. 2015). This method operates under a “limiting factor” approach, weighing HSI estimates as “low” if any of the variables included in the model has a low score. Ecological layer weights were informed by Yan et al. (2021), with shallow shelf area (25.0%), mangrove extent (14.8%), and estuarine discharge (3.1%) as the most influential environmental predictors of global *P. pristis* occupancy and extinction. These scores were normalized to maintain their relative contributions in a HSI model based solely on ecological variables (i.e., excluding human pressures or management‐related measures), resulting in a proportional weighting ratio of 58:35:7 for shelf, mangrove, and flow accumulation layers, respectively.

Sawfish records from López‐Angarita, Cubillos‐M, et al. (2021) were incorporated into the HSI to represent local spatial density and observed habitat use. This KDE‐based layer included historical sightings from 2000 to 2015. The KDE‐based layer potentially introduces biases due to spatial and temporal gaps in documented sawfish reports which are influenced by encounter likelihood, fishing pressure, population density, site accessibility, and the lack of a centralized reporting platform. To account for these limitations, four weighted scenarios to explore the trade‐off between prioritizing ecological factors and incorporating historical human observations (i.e., KDE) were developed to evaluate overall *P. pristis* habitat suitability in Panama and Colombia in the Eastern Tropical Pacific. In Scenario 1, baseline (ecological variables only) was defined by assigning 0% weight to the KDE layer, relying solely on the three ecological variables weighted by the ratio of 58:35:7 for shelf, mangrove, and flow accumulation layers, respectively. Scenarios 2, 3, and 4 sequentially introduced and increased the influence of historical observations by assigning the KDE layer a weight of 10%, 20%, and 40%, respectively, with the remaining weight distributed proportionally among the three ecological variables.

HSI outputs for each scenario were evaluated by performing an Uncertainty Analysis via a Monte Carlo simulation with 1000 iterations (Zajac et al. 2015). All input ecological variables were “disrupted” within a uniform distribution range of ±20% around their scores, while the KDE layer was disrupted within ±50% to account for input data uncertainty propagation through the scenario models (Zajac et al. 2015). River basins (*N* = 112) were classified into habitat suitability categories (High, Moderate, or Low) for *P. pristis* based on the outputs of the HSI and the Uncertainty Analysis. This classification considered the mean HSI score, calculated as the mean across all 500 m resolution cells within the basin. The latter was defined as how frequently a basin was classified as High across all 1000 Monte Carlo iterations. A global HSI threshold was defined as the overall mean HSI plus one standard deviation (SD) across all scenarios combined. This conservative threshold was estimated from the HSI distribution to avoid imposing arbitrary fixed cutoffs and reduce the uncertainty propagation in the spatial assignment of habitat classifications. The threshold was used to identify basins representing the upper tail of habitat suitability distribution, thereby focusing on areas most likely to function as core habitat for *P. pristis* . Basins exceeding this threshold in at least 75% of iterations were treated as consistently High under uncertainty. Habitat suitability and sampling sites were then overlaid with Important Shark and Ray Areas (ISRAs; https://sharkrayareas.org) delineated for Pacific Panama and Colombia (García‐Rodríguez et al. 2025) to examine congruence.

## 3 Results

### 3.1 eDNA Assay Validation

**FIGURE 2:**
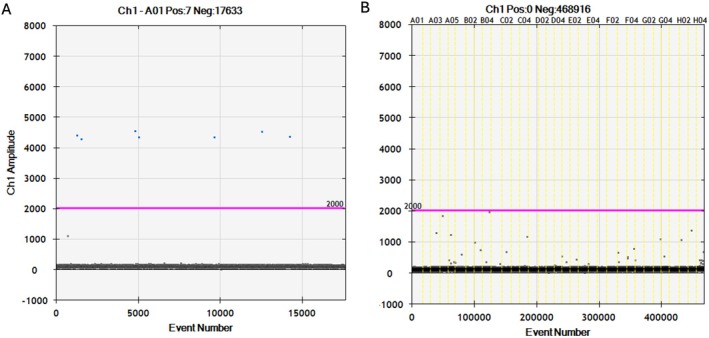
Droplet Digital PCR results of in vitro assay validation. In each plot, droplets are classified as positive (blue) or negative (gray) for Largetooth Sawfish, *Pristis pristis* , DNA based on a manual threshold (pink line) of 2000 relative fluorescence units (RFUs) above the negative band, detected using a Bio‐Rad QX200 Droplet Reader, QuantaSoft software, and Rare Event Detection analysis. (A) Successful amplification of *P. pristis* DNA using 0.2 × 10^−7^ ng genomic DNA, with seven positive droplets in a single replicate and a target DNA concentration of 0.47 copies/μL. (B) No amplification of DNA in eight exclusion species (Table 3), in triplicate reactions, with zero positive droplets. In B, wells are separated by yellow vertical lines.

The ddPCR eDNA assay successfully amplified DNA from all *P. pristis* during in vitro assay validation (Figure 2A), spanning individuals from Bangladesh, Papua New Guinea, Australia, Nicaragua, Panama, Ecuador, Peru, Brazil, the United States, and The Gambia. The positive droplet range for the assay was defined as 4000–6000 RFUs and the MT was conservatively set to 2000 RFUs, with the highest amplitude of droplets in the NTC reactions being ~1500 RFUs. Droplets between 2001 and 3999 RFUs were therefore considered to be above the MT, but not within the known positive range for the species. The LoD for the assay was determined to be 0.07 copies/μL, reflecting the average concentration of a one‐droplet detection.

In silico assay specificity testing revealed that the cumulative number of bp differences in the primers and probe sequences ranged from 7 to 14 across 15 exclusion species. This testing included all three nontarget *Pristis* spp. and seven other (non‐*Pristis*) genera from four of the five rhino ray families (Pristidae, Rhinidae, Glaucostegidae, Rhinobatidae, see Table 2). During in vitro cross‐testing, none of the ddPCR replicates for the eight representative exclusion species, including all four other sawfish species and four *Pseudobatos* spp., showed any amplification (i.e., met zero of the data analysis criteria, Figure 2B). This confirms the assay is species‐specific in the Eastern Pacific and Western Atlantic (Figure 2B), with high likelihood of range‐wide assay specificity.

### 3.2 eDNA Surveys

Across all sampling sites, waters were warm (mean = 27.17°C, SD = 0.71) and relatively shallow, with a mean sampling depth of 2.1 m (SD = 0.28). Sites sampled in the Gulf of Chiriquí were slightly shallower on average than those in the Darién and Chocó regions (Table 4). Mean salinity for sampling sites in the Gulf of Chiriquí was 21.56 (SD = 10.32), although salinities ranged from zero (freshwater) to 33, and waters were moderately to highly turbid (average secchi depth of 86.35 cm) (Table 4).

**TABLE 4:** Mean (standard deviation) environmental data collected at water sampling sites within each region of Panama and Colombia during environmental DNA surveys for Largetooth Sawfish, *Pristis pristis* .

Country	Region	*N*	Sampling depth (m)	Temperature (°C)	Salinity	Turbidity (cm)
Panama	Gulf of Chiriquí	48	1.76 (1.20)	27.76 (1.05)	21.56 (10.32)	86.35 (10.52)
Panama	Darién	18	2.26 (1.00)	27.36 (1.08)	NA	NA
Colombia	Choco	33	2.23 (1.02)	26.38 (1.31)	NA	NA

Abbreviation: NA, not available.

Total eDNA extracts from field samples were high molecular weight DNA with a mean concentration of 115.07 ng/μL (SD = 12.63). None of the negative controls (field, extraction, ddPCR) met any of the analysis criteria, indicating that contamination by exogenous *P. pristis* DNA was unlikely during any stage or day of sample processing. None of the eDNA samples collected at 99 sites spanning marine, estuarine, and freshwater habitats in Panama and Colombia met any of the three analysis criteria for positive detections of *P. pristis* DNA, including the replicate samples treated with BSA (Figure 1).

### 3.3 Habitat Suitability Index Model

**FIGURE 3:**
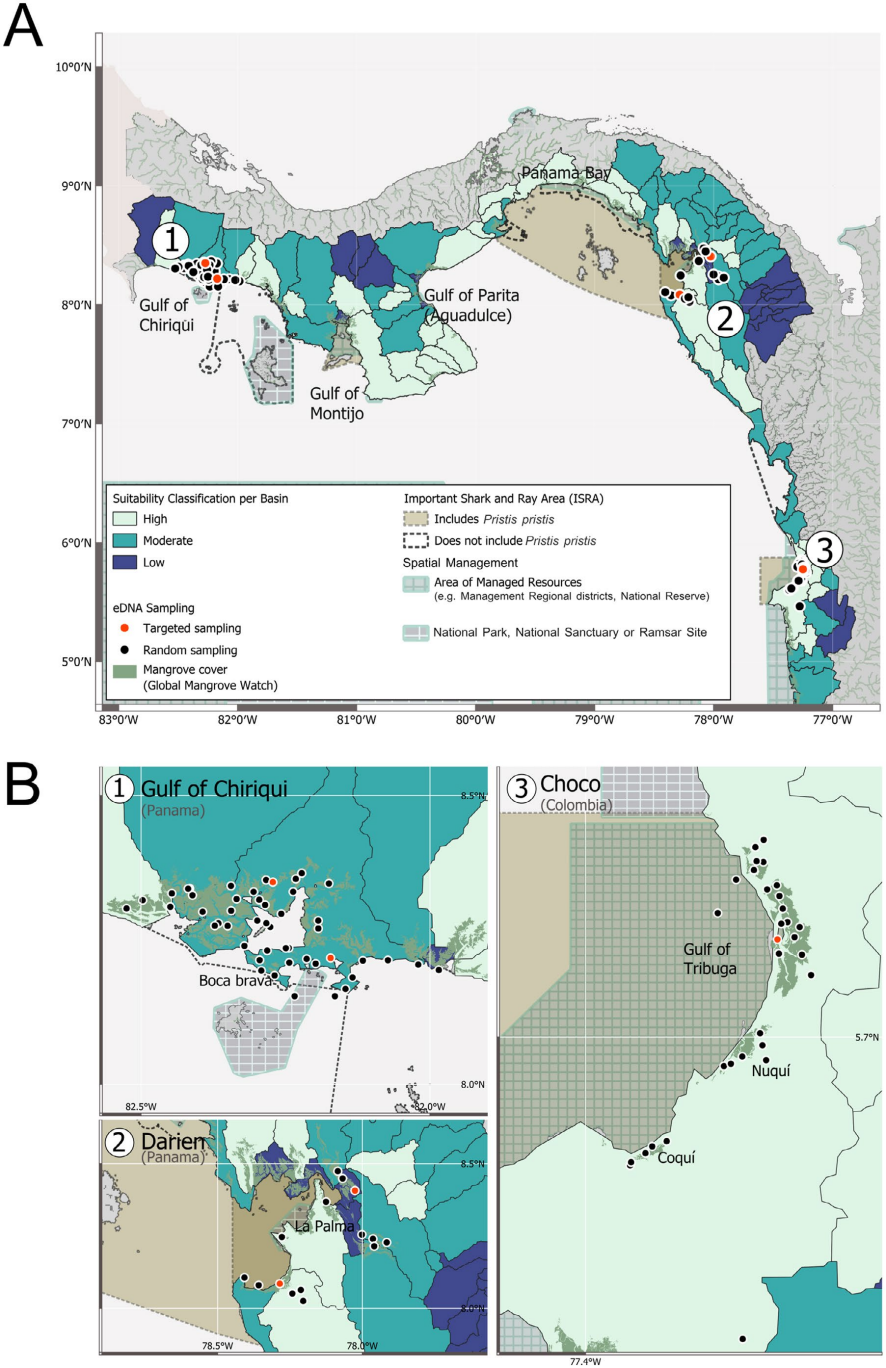
Basin‐level habitat suitability for Largetooth Sawfish, *Pristis pristis*, under the Habitat Suitability Index (HSI) model baseline scenario (S1) in the Eastern Tropical Pacific. (A) Habitat suitability across 112 Pacific basins in Panama and Colombia, derived from the weighted geometric mean HSI. Three sampling regions in Panama (1, Gulf of Chiriquí; 2, Darién) and Colombia (3, Chocó). (B) Environmental DNA (eDNA) sampling sites (circles) per region and their adjacent basins. River basins are color‐coded by suitability category, with lighter hues indicating higher suitability. Mangrove cover (green; from Global Mangrove Watch; https://www.globalmangrovewatch.org), spatial management (gridded polygons), and Important Shark and Rays Areas (ISRAs; dashed lines) are shown, with ISRAs where *P. pristis* is a Qualifying Species highlighted.

*Pristis pristis* habitat suitability was modeled across 67,810km^2^ of the Pacific drainage of Panama (51,466 km^2^) and the Chocó region of Colombia (16,344 km^2^). Under the four scenarios, increasing the weight of the KDE layer reduced the predicted area of High habitat suitability (Table 5). Of the 112 river basins evaluated, the baseline model (Scenario 1), which focused solely on ecological variables, yielded a large continuous area of 55,988 km^2^ of Moderate and High habitat suitability basins, corresponding to 82.6% of the total evaluated area in Panama and Colombia. Within each country, the High category alone represented 35.72% of the evaluated basin area in Panama and 24.3% in Colombia (Figure 3 and Table 5). In Scenarios 2, 3, and 4, where KDE acted as a limiting factor in the WGM model, the total area of Moderate and High suitable habitat fell sharply. This reduction represents a decline in suitable habitat to 506 km^2^ (0.75%) in Scenario 2 and 399.5 km^2^ (0.59%) in Scenario 3, with 0 km^2^ (0%) of suitable habitat in Scenario 4 (Figures S1 and S2; Table 5). The Uncertainty Analysis using Monte Carlo permutations yielded a consistently narrow distribution of HSI scores for basin‐mean HSI scores under each scenario (Table 5 and Figure S1). The mean HSI scores for the baseline model (Scenario 1) ranged from 0.611 to 0.819, while Scenario 4 (40% KDE) showed the widest within‐scenario dispersion, ranging from 0.047 to 0.814. Similarly, the median confidence interval (CI) widths remained low in all scenarios, indicating that most basins had narrow uncertainty even under increased model constraints. Dispersal of the 95% CI widths increased with the percentage of KDE weight. Scenario 1 (0% KDE) showed CI ranges from 0.180 to 0.236, while Scenario 4 (40% KDE) showed the largest overall dispersal, with CI ranges from 0.007 up to a maximum of ∼0.368. However, when aggregating the results across scenarios, the overall variances increase considerably as HSI estimates were skewed towards Scenario 1.

**TABLE 5:** Habitat Suitability Index (HSI) and suitable river basin area for Largetooth Sawfish, *Pristis pristis* , in Pacific Panama and Colombia across four modeling scenarios with increasing Kernel Density Estimation (KDE) weight.

Scenario	KDE weight	Mean HSI score range	Total area of moderate and high suitability (km^2^)	% of High suitability	
				Panama	Colombia
S1 (baseline)	0	0.635–0.819	55,987.94	35.72	24.3
S2	10	0.322–0.791	506.41	0	2.44
S3	20	0.165–0.785	399.54	0	2.44
S4	40	0.047–0.814	0	0	0

*Note:* Mean HSI is the range of scores across river basins (*N* = 112). The total suitable basin area encompasses the area categorized as Moderate or High suitability, based on global HSI and probability thresholds. The percentage of basin area categorized as High suitability is reported separately for Panama and Colombia.

**FIGURE 4:**
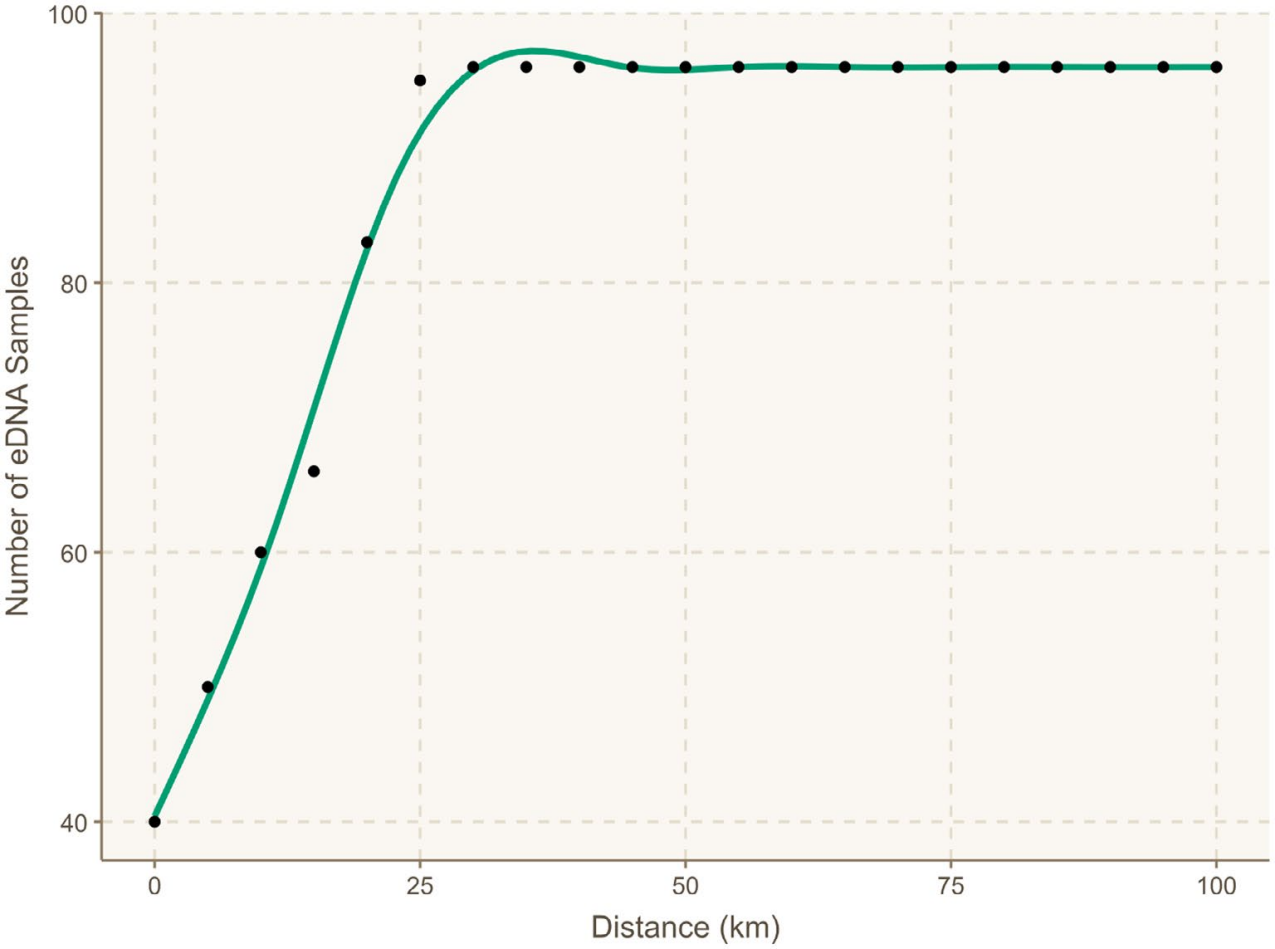
Cumulative proximity curve of environmental DNA (eDNA) sampling sites relative to river basins categorized as having High Habitat Suitability Index (HSI) scores for Largetooth Sawfish, *Pristis pristis* , under Scenario 1. The curve represents the cumulative number of samples within increasing distance from high‐suitability basins (from 0 to 100 km with each black dot representing 5 km).

The drainage basins encompassing the eDNA sampling sites in the Gulf of Chiriquí, Darién, and Gulf of Tribugá regions consistently showed overall high mean HSI scores across all four scenarios (Figures S1 and S2). Of the 99 eDNA sampling sites, 42% (*N* = 40) were located directly within basins with High suitability under Scenario 1, the baseline model (Figure 3). The remaining 59 sampling sites were situated outside these basins but were within 25 km of a High suitability basin (Figures 3 and 4).

## 4 Discussion

### 4.1 Range‐Wide eDNA Assay for *Pristis prisits*

This study developed a novel ddPCR eDNA assay to support highly sensitive, range‐wide global surveys for *P. pristis* to identify priority locations for conservation actions. This assay was validated in vitro, successfully amplifying DNA from individuals from all four regions the species once occurred: the Eastern Pacific, Western Atlantic, Eastern Atlantic, and Indo‐West Pacific. Assay functionality across the global range of a species is highly advantageous, creating time and cost efficiencies while improving the comparability of data from independent surveys in different regions. Assay validation using individuals from across the range of a target species is especially critical for species with a high degree of population structure like *P. pristis* (Phillips et al. 2011; Feutry et al. 2015). Population‐level mutations in primer/probe annealing regions can negatively impact PCR amplification and/or efficiency, increasing the risk of false negatives (e.g., Endo et al. 2024; Rodrigues et al. 2025). Owing to female philopatry to freshwater rivers for parturition, *P. pristis* exhibits matrilineal structure along coastlines and high divergence across ocean basins (Phillips et al. 2011; Faria et al. 2013; Feutry et al. 2015). The ability of our novel ddPCR eDNA assay to detect *P. pristis* across all four regions it once occurred despite population‐level differences offers broad geographic utility and negates concerns of false negatives stemming from the use of an assay designed for only a specific location/population (e.g., Simpfendorfer et al. 2016; Cooper et al. 2021; Rodrigues et al. 2025).

Comprehensive cross‐testing of a species‐specific assay with exclusion species is required prior to conducting eDNA field surveys (Wilcox et al. 2013). The ddPCR eDNA assay developed in this study was demonstrated to be species‐specific both in silico and in vitro in the Eastern Pacific and Western Atlantic. It is also anticipated to be species‐specific across the global range of *P. pristis* . In silico testing revealed numerous cumulative bp mismatches in the primer and probe regions of exclusion species spanning four of the five families of rhino ray, and in vitro testing did not result in any cross‐amplification in any other species of sawfish, nor representative *Pseudobatos* guitarfishes. Given these findings, the assay is not anticipated to cross‐amplify DNA from species in the more distantly related exclusion species of the remaining family (banjo rays, Trygonorrhindae) or genera of non‐sawfish rhino rays that mtDNA 12S rRNA sequence data were not available for assay validation. Regardless, we recommend further confirmation of assay specificity using tissue‐derived DNA from additional exclusion species that co‐occur in the Eastern Atlantic and Indo‐West Pacific prior to its use in eDNA surveys in these regions to achieve higher assay validation standards, as described by Thalinger et al. (2021). This is particularly important for eDNA surveys using digital PCR since samples are “consumed” during the droplet reader stage of analysis. This makes sequencing positive amplicons an unreliable option to confirm species identity. Replicate samples can be run in parallel and sequenced after PCR cycling (and before droplet reader analysis, see Baker et al. 2018), however sequencing single‐copy amplicons is challenging and each PCR reaction contains a unique suite of eDNA, which may not consistently contain low‐copy target DNA.

The integration of digital PCR technology into eDNA surveys is the most promising approach for detecting exceptionally rare species from environmental samples (see Wood et al. 2019; Lehman et al. 2022). When robustly designed and tested, digital PCR offers precise quantification estimates of target DNA, with the ability to reliably detect a single copy of target DNA. Prior to this study, eDNA surveys for Smalltooth Sawfish, *Pristis pectinata,* in the United States integrated ddPCR technology (Lehman et al. 2022), however, other species‐specific in situ eDNA surveys for sawfishes have primarily relied on conventional (cPCR) or quantitative PCR (qPCR) platforms (Simpfendorfer et al. 2016; Cooper et al. 2021; Bonfil et al. 2024; Valerio‐Vargas et al. 2025). cPCR and qPCR platforms are predisposed to false negatives due to less sensitive detection capabilities and lack precision in quantification of target DNA (Mauvisseau et al. 2019; Wood et al. 2019; Guri et al. 2024). cPCR and qPCR platforms are also more prone to PCR inhibitors, such as tannins that are often present in highly vegetative aquatic habitats, including mangroves (Dingle et al. 2013; Lance and Guan 2020). A ddPCR eDNA assay recently designed specifically for *P. pristis* populations in Brazil (Rodrigues et al. 2025) failed to incorporate cross‐testing in exclusion species, or a quantitative assessment of assay sensitivity (i.e., LoD), thus limiting both its wider geographic utility and “readiness” of the assay for in situ use (e.g., Thalinger et al. 2021).

The ability of digital PCR to detect a single copy of target DNA heightens concerns of contamination, particularly by aerosolized DNA. A high level of rigor is required in both the field and laboratory during eDNA surveys using digital PCR technology to ensure positive detections are authentic, rather than contamination by exogenous DNA (see Sepulveda et al. 2020). We recommend that eDNA survey data that use digital PCR be independently evaluated for rigor by end‐users before integration into conservation planning as an additional safeguard against false positive data. Sample collection and processing protocols should be highly detailed, fully transparent, and results of all negative controls clearly reported. In this study, we outlined strict contamination prevention controls used during each stage of sample processing, with thorough testing for contamination. Screening of field, DNA extraction, and ddPCR negative controls suggested no contamination of exogenous *P. pristis* DNA occurred during this study.

### 4.2 eDNA Surveys in the Eastern Tropical Pacific

Despite the use of the most sensitive technology available, eDNA surveys in the Darién, Chiriquí, and Chocó regions of the Eastern Tropical Pacific did not detect the presence of *P. pristis* . This suggests their absence near the sampling sites during surveys, although the interpretation of non‐detections requires caution.

Non‐detections imply that DNA was either not captured in the field or was not detectable during laboratory analysis (see Lehman et al. 2022). Failure to capture target DNA during field surveys can indicate the absence of the species in the vicinity of the sampling site, but can also stem from the collection of an insufficient volume of water, an inadequate sampling regime, or environmental factors such as flow regime and eDNA degradation rate which influence the transport and lifespan of eDNA in the environment (Rees et al. 2014; Collins et al. 2018; Nagarajan et al. 2022). The failure to detect captured DNA during laboratory analysis is typically associated with assay performance and sensitivity, the presence of PCR‐inhibitors, and/or sub‐sampling biases where only a portion of the total sample is screened for target species DNA. Here, 3 L of water was collected at each site, which has previously been shown to be sufficient for capturing sawfish DNA despite low abundance (see Lehman et al. 2022; Valerio‐Vargas et al. 2025). Samples were analyzed using a thoroughly validated, highly sensitive ddPCR eDNA assay, with no evidence of PCR‐inhibition, although only 10% of the total eDNA extract was screened for *P. pristis* DNA.

The lack of positive detections during field surveys likely reflects the rarity of *P. pristis* in the sampling regions combined with limited sampling effort, exacerbated by environmental factors. The inability to detect *P. pristis* DNA at sampling sites could reflect spatial (e.g., proximity to individuals) and/or temporal (e.g., timing of presence vs. sampling) mismatches between eDNA sampling and species presence (Rees et al. 2014). These tropical regions are characterized by highly dynamic, tidally influenced environments, which present challenges for the successful capture of highly diluted, low copy target DNA from rare species. Lifespans of eDNA during surveys would likely have been limited to several hours due to warm water temperatures, high organic matter discharge, microbial activity, and UV exposure (Collins et al. 2018; Nagarajan et al. 2022). In such environments, when target species abundance is low, spatially comprehensive sampling regimes are needed, ideally with seasonal and/or annual replication to support robust interpretations of species presence/absence. While the spatial coverage of eDNA sampling was relatively comprehensive in the Gulf of Chiriquí, surveys were conducted in a two‐week time period of a single year. Fewer samples were collected in each of the Darién and Chocó and sites were localized, focusing on accessible areas thought to most likely harbor *P. pristis* . *Pristis pristis* may still occur in low abundances in these regions but could have evaded detection if individuals were not in the immediate vicinity of sampling sites, especially given the probable short eDNA lifespans and high flow rates in these dynamic systems that would have rapidly diluted and transported low‐copy eDNA away from their source.

Adult and juvenile *P. pristis* were historically prevalent in the Darién, Chiriquí, and Chocó regions of the Eastern Tropical Pacific, with contemporary records from 2000 to 2015 and 2022 suggesting the persistence of small numbers (López‐Angarita, Cubillos‐M, et al. 2021; IUCN SSC Shark Specialist Group 2023). No sawfishes were reported concurrently during eDNA surveys, although the 2022 record was of a young‐of‐the‐year sawfish (~80 cm total length) reported on social media in the Tuira River, Chepigana, in the Darién (Instagram 2022). This sawfish was reported in August, just one month after eDNA surveys in this region, and in an area that lacked comprehensive eDNA sampling coverage. Future eDNA surveys in these regions should prioritize greater sampling coverage with temporal replication to overcome some of the limitations of the current study. The use of passive eDNA samplers should also be considered owing to their ability to capture eDNA in flowing waters for an extended period, potentially increasing the likelihood of detection where *P. pristis* is present (Chen et al. 2024). This sampling approach would come with additional challenges, such as the logistics of the deployment and retrieval of samplers in highly dynamic tropical environments.

The probabilities of detecting target DNA under natural conditions should be evaluated to support future eDNA monitoring surveys (e.g., Wilcox et al. 2016). Environmental variability, habitat complexity, and species ecology all influence eDNA detection probability, and estimating this empirically through repeated field sampling is needed to support interpretation of non‐detection data (see Tingley et al. 2021). Field‐derived detection probabilities would allow for the parameterization of more realistic species distribution and occupancy models that integrate both methodological and ecological uncertainties as eDNA becomes more routinely incorporated into monitoring efforts.

### 4.3 Habitat Suitability Index

The HSI served as an independent perspective on potential habitat for *P. pristis* in the region, incorporating key environmental variables across its life stages (Zajac et al. 2015; Yan et al. 2021). Global models evaluating extinction risk in all five species of sawfish previously validated the relevance of shallow shelf areas (< 25 m depth), mangrove coverage, and discharge rate (i.e., freshwater input) as key predictors of sawfish occurrence and ecological carrying capacity (Yan et al. 2021). Here, a posteriori remote sensing‐derived HSI modeling combined with eDNA surveys provides a complementary approach for identifying promising habitats in data‐limited areas, despite their differing spatial and temporal scales. Remote sensing informs the spatial extent of the environment, seasonal variations, and hydrological regimes that enhance predictive models, while eDNA offers finer‐scale snapshots of species occurrence (Wang et al. 2024; Zong et al. 2024).

The baseline model (Scenario 1) identified basins with high mean HSI scores in key regions, including basins across the Gulf of Montijo, the Gulf of Parita, and the Darién region in Panama and the Gulf of Tribugá in Colombia. These basins overlapped with several ISRAs which were delineated for *P. pristis* (among other species), namely the Gulf of San Miguel and Tuira River ISRA (Darién region) and the Gulf of Montijo ISRA in Panama, and the Tribugá Gulf ISRA in Colombia (García‐Rodríguez et al. 2025). The Gulf of Chiriquí consistently showed mean HSI above the 50% quantile across all scenarios, with basins of Moderate and adjacent High suitability. The decline in predicted suitable habitat under scenarios with higher KDE weight does not necessarily indicate habitat loss. Rather, KDE‐constrained models restrict suitable habitat categories to locations with documented observations, highlighting potential reporting gaps in otherwise ecologically suitable areas. Across scenarios, predicted suitable habitats showed strong spatial alignment with eDNA sampling sites, with all water samples collected within, or in proximity to, basins with highly suitable habitat. This suggests that eDNA non‐detections are unlikely to be driven by sampling in unsuitable habitats and instead reflect spatiotemporal limitations inherent to sampling water in dynamic environments and/or the absence of *P. pristis* near sampling sites during eDNA surveys.

The development of four scenarios which incrementally increased the weight of historical sightings acted as a relevant internal sensitivity test. The results showed the greater reliance on KDE severely reduced the extent of predicted suitable habitat, likely a result of reporting gaps and observation bias rather than ecological unsuitability (López‐Angarita, Cubillos‐M, et al. 2021; Zajac et al. 2015). The WGM models were structurally robust as reflected in the narrow uncertainties and not highly sensitive to moderate perturbations in the input layers. Despite the KDE models having a smaller 95% CI, this possibly stems from two factors. First, KDE acts as a strong limiting factor with increasing weight, as more drainage basins are excluded due to lack of historical sightings, reducing the number of raster cells that contribute to the global HSI scores and CI estimates. Second, increasing KDE weight constrains the variation from iteration to iteration and skews the confidence interval to lower scores. Thus, the baseline model captures potential habitat, while the KDE‐weighted scenarios reflect realized habitat under data limitations. Models incorporating historical sightings in data‐poor regions may underrepresent suitable habitat and should be interpreted conservatively.

HSI models simultaneously provided an opportunity to assess the alignment of ecologically relevant habitats for *P. pristis* to the ISRAs delineated in the Eastern Tropical Pacific (https://sharkrayareas.org/). The results highlight the Darién region, including the Gulf of San Miguel and Tuira River ISRA in Panama and the Tribugá Gulf ISRA in Colombia as areas with multiple basins having high suitability habitat under the baseline model. These results indicate the relevance of these ISRAs for sawfish conservation and are consistent with the previous designation of these areas as “bright‐spots” for *P. pristis* (López‐Angarita, Cubillos‐M, et al. 2021). The Gulf of Chiriquí ISRA, which encompasses Coiba National Park, and the Panama Muertos Bay ISRA do not currently include *P. pristis* as “Qualifying Species” (species that meet the ISRA Criteria) due to a lack of data supporting their regular contemporary occurrence. Although eDNA surveys did not reveal detections of *P. pristis* in this region, several basins in the area with high suitability scores were identified, suggesting their potential ecological value for this species and their relevance for ongoing monitoring efforts.

### 4.4 Conservation Implications

Monitoring rare and elusive species in data‐poor regions presents major challenges, where multiple lines of evidence are often needed to inform conservation priorities and actions. To date, resolving uncertainties of where sawfishes persist in remote regions have largely relied on local ecological knowledge obtained through interviews (e.g., Leeney and Poncelet 2015; Chowdhury et al. 2018). We recommend eDNA surveys using digital PCR technology be conducted in nations where presence of sawfishes remains uncertain, offering the best chances of detection where abundances are low. Owing to its greater sensitivity, eDNA approaches using digital PCR support time and cost efficiencies compared to traditional survey methods while negating the need to capture and handle threatened species. Where numerous species of interest co‐occur, digital PCR assays can be designed to simultaneously detect multiple species in a single analysis, further improving cost and time efficiencies (e.g., Dias et al. 2025).

Range‐wide eDNA surveys using the highly sensitive ddPCR eDNA assay developed here combined with temporally and spatially robust sampling regimes that incorporate HSIs would more definitely address unknowns surrounding where *P. pristis* persists, and where local extinctions may have occurred. This would allow for more effective prioritization of conservation initiatives, directing resources to where they are most urgently needed, and where *P. pristis* are confirmed to persist. eDNA surveys in the Darién and Chiriquí regions of Panama and northern Chocó region of Colombia failed to detect *P. pristis* despite the presence of highly suitable habitat. These surveys also included sampling sites within areas designated as ISRAs for this species in the Darién region of Panama and the Gulf of Tribugá in Colombia, as well as proximity to protected areas (e.g., Utría National Natural Park, Chocó) and areas of community‐based management where human activities are restricted. The loss of mangrove cover on the Pacific coasts of Panama and Colombia has been relatively low since 2000; however, coastal developments, land‐use changes, and ongoing artisanal and small‐scale fisheries continue to threaten *P. pristis* (López‐Angarita et al. 2018; Mejía‐Rentería et al. 2018; López‐Angarita, Villate‐Moreno, et al. 2021). Despite these ongoing threats, bright spots of sawfish persistence across their historical range occur in the Eastern Tropical Pacific and globally, providing suitable candidates for spatially and temporally comprehensive eDNA surveys using our novel assay.

## Author Contributions

**Juan C. Cubillos‐M:** conceptualization (lead), data curation (lead), formal analysis (lead), funding acquisition (lead), investigation (lead), methodology (equal), resources (equal), software (equal), visualization (lead), writing – original draft (equal), writing – review and editing (equal). **Annmarie Fearing:** data curation (equal), formal analysis (equal), investigation (equal), methodology (equal), validation (equal), visualization (supporting), writing – review and editing (equal). **Demian D. Chapman:** conceptualization (lead), funding acquisition (lead), methodology (equal), project administration (equal), resources (equal), writing – original draft (equal), writing – review and editing (equal). **Peter M. Kyne:** investigation (equal), methodology (equal), visualization (supporting), writing – original draft (equal), writing – review and editing (equal). **P. Joana Dias:** investigation (equal), methodology (equal), writing – review and editing (equal). **Cindy Gonzalez:** investigation (equal), methodology (equal), writing – review and editing (supporting). **Ryan N. Lehman:** investigation (equal), methodology (equal), writing – review and editing (supporting). **Yaliana Chichaco:** investigation (equal), methodology (equal), validation (equal), writing – review and editing (supporting). **Bryan L. Huerta‐Beltrán:** data curation (equal), formal analysis (equal), investigation (equal), methodology (equal), validation (equal), writing – review and editing (equal). **Kayla L. McCulloch:** formal analysis (equal), investigation (equal), validation (equal), writing – original draft (supporting), writing – review and editing (supporting). **Sarah M. Toepfer:** data curation (equal), investigation (equal), methodology (equal), validation (equal), writing – review and editing (supporting). **John K. Carlson:** conceptualization (lead), funding acquisition (lead), investigation (equal), methodology (equal), resources (equal), writing – original draft (equal), writing – review and editing (equal). **Nicole M. Phillips:** conceptualization (lead), data curation (equal), formal analysis (lead), funding acquisition (lead), investigation (lead), methodology (lead), project administration (lead), resources (lead), software (lead), supervision (lead), validation (lead), visualization (equal), writing – original draft (lead), writing – review and editing (lead).

## Funding

Assay design and eDNA surveys in the Chiriquí region were supported via funding from the Islas Secas Foundation to NMP, JKC, and DDC. Funding for eDNA surveys in the Darién region and Colombia was provided by the Save Our Seas Foundation (Small Grant no. SOSF 459) and Carl von Ossietzky University of Oldenburg to JCCM. This research was supported by The University of Southern Mississippi and the Mississippi INBRE, funded by an Institutional Development Award (IDeA) from the National Institute of General Medical Sciences of the National Institutes of Health under grant number P20GM103476. The scientific results and conclusions, as well as any views or opinions expressed herein, are those of the author(s) and do not necessarily reflect those of institutions or data providers.

## Ethics Statement

Biological samples were collected, shipped, and stored under Endangered Species Act Permit numbers 20590 and 27294. Tissue samples were imported under CITES Certificate of Scientific Exchange (COSE) 15US134901/9, registration number US179, issued to The University of Southern Mississippi Gulf Coast Research Laboratory Museum, and exported under CITES Registration numbers GB003 (National Museums Scotland) and AU004 (Museum and Art Gallery of the Northern Territory, Australia). Access to sampling locations in Colombia was granted by the General and local community councils, Consejo Comunitario General Los Riscales (Nuquí) and in collaboration with MarViva Foundation. In Panama, access and field activities within the Punta Patiño Ramsar site were authorized and supported by the NGO National Association for Nature Conservation (ANCON), which manages the Punta Patiño RAMSAR site and protected area.

## Conflicts of Interest

The authors declare no conflicts of interest.

## Supporting information


**Figure S1:** Basin‐mean HSI (blue hue areas) and 95% Confidence Interval (red hue areas) of Habitat Suitability Index (HSI) scores per river basin for all four modeled scenarios for Largetooth Sawfish, *Pristis pristis*. Scenario 1—Baseline: Only ecological relevant variables. Scenario 2: Kernel Density Estimation (KDE) 10% variable weight in the HSI‐weighted geometric mean (WGM) model. Scenario 3: KDE 20%. Scenario 4: KDE 40%.
**Figure S2:** Habitat suitability classification for all scenarios. River basins were classified into *High*, *Moderate*, or *Low* suitability for Largetooth Sawfish, *Pristis pristis* , based on the mean Habitat Suitability Index (HSI) and the probability of exceeding a global threshold calculated from Monte Carlo simulations. (A) Scenario 1—Baseline: Only ecologically relevant variables. (B) Scenario 2: Kernel Density Estimation (KDE) 10% variable weight in the HSI‐weighted geometric mean (WGM) model. (C) Scenario 3: KDE 20%. (D) Scenario 4: KDE 40%.

**Table S1:** Water collection and filtration data for *Pristis pristis* enviornmental DNA surveys in Panama and Colombia.
**Table S2:** Raw Droplet Digital PCR data for *Pristis pristis* environmental DNA surveys in Panama and Colombia.

## Data Availability

Environmental DNA collection and ddPCR data are available as Tables S1 and S2 in Supporting Information. All remote sensing information and spatial layers used for HSI are accessible through their source project and Open access.

## References

[ece373733-bib-0001] Altschul, S. F. , W. Gish , W. Miller , E. W. Myers , and D. J. Lipman . 1990. “Basic Local Alignment Search Tool.” Journal of Molecular Biology 215: 403–410. 10.1016/S0022-2836(05)80360-2. 2231712

[ece373733-bib-0002] Baker, C. S. , D. Steel , S. Nieukirk , and H. Klinck . 2018. “Environmental DNA (eDNA) From the Wake of the Whales: Droplet Digital PCR for Detection and Species Identification.” Frontiers in Marine Science 5: 133. 10.3389/fmars.2018.00133.

[ece373733-bib-0003] Bonfil, R. , P. Díaz‐Jaimes , P. Palacios‐Barreto , O. U. Mendoza Vargas , and M. Ricaño‐Soriano . 2024. “Improved eDNA Assay Evidences Further Refugia for Critically Endangered Smalltooth Sawfish ( *Pristis pectinata* ) in Mexico.” Frontiers in Marine Science 11: 1290661. 10.3389/fmars.2024.1290661.

[ece373733-bib-0004] Capo, E. , G. Spong , S. Koizumi , et al. 2021. “Droplet Digital PCR Applied to Environmental DNA, a Promising Method to Estimate Fish Population Abundance From Humic‐Rich Aquatic Ecosystems.” Environmental DNA 3: 343–352. 10.1002/edn3.115.

[ece373733-bib-0005] Chen, X. , S. Li , J. Zhao , and M. Yao . 2024. “Passive eDNA Sampling Facilitates Biodiversity Monitoring and Rare Species Detection.” Environment International 187: 108706. 10.1016/j.envint.2024.108706. 38696978

[ece373733-bib-0006] Chowdhury, G. W. , S. Sabbir , and A. B. Haque . 2018. “Recent Records of Large Tooth Sawfish *Pristis pristis* (Linnaeus, 1758) From Parerhat of Pirojpur District in the Southwestern Bangladesh.” Bangladesh Journal of Zoology 46: 255–262. 10.3329/bjz.v46i2.39057.

[ece373733-bib-0007] Claver, C. , M. Bhendarkar , I. Mendibil , et al. 2026. “Lessons Learned From Applying eDNA Surveying to Diadromous Fish Detection Across the Northeast Atlantic Region.” Journal of Fish Biology: 1–15. 10.1111/jfb.70413. PMC13397221 41992080

[ece373733-bib-0008] Collins, R. A. , O. S. Wangensteen , E. J. O'Gorman , S. Mariani , D. W. Sims , and M. J. Genner . 2018. “Persistence of Environmental DNA in Marine Systems.” Communications Biology 1: 185. 10.1038/s42003-018-0192-6. 30417122 PMC6218555

[ece373733-bib-0009] Cooper, M. K. , R. Huerlimann , R. C. Edmunds , et al. 2021. “Improved Detection Sensitivity Using an Optimal eDNA Preservation and Extraction Workflow and Its Application to Threatened Sawfishes.” Aquatic Conservation: Marine and Freshwater Ecosystems 31: 2131–2148. 10.1002/aqc.3591.

[ece373733-bib-0010] Dias, P. J. , R. N. Lehman , B. L. Huerta‐Beltrán , et al. 2025. “A Novel ddPCR Assay for eDNA Detection and Quantification of Greater Amberjack *Seriola dumerilli* and Three Congeners in US Waters: Challenges and Application to Fisheries Independent Surveys.” PeerJ 13: e18778. 10.7717/peerj.18778. 39886022 PMC11781265

[ece373733-bib-0011] Dingle, T. C. , R. H. Sedlak , L. Cook , and K. R. Jerome . 2013. “Tolerance of Droplet‐Digital PCR vs Real‐Time Quantitative PCR to Inhibitory Substances.” Clinical Chemistry 59: 1670–1672. 10.1373/clinchem.2013.211045. 24003063 PMC4247175

[ece373733-bib-0012] Endo, N. , Y. Nihei , T. Fujita , et al. 2024. “Explaining the Impact of Mutations on Quantification of SARS‐CoV‐2 in Wastewater.” Scientific Reports 14: 12482. 10.1038/s41598-024-62659-y. 38816525 PMC11139995

[ece373733-bib-0013] Espinoza, M. , R. Bonfil‐Sanders , J. Carlson , et al. 2022. “ *Pristis pristis* .” The IUCN Red List of Threatened Species 2022: e.T18584848A58336780. 10.2305/IUCN.UK.2022-2.RLTS.T18584848A58336780.en.

[ece373733-bib-0014] Faria, V. V. , M. T. McDavitt , P. Charvet , T. R. Wiley , C. A. Simpfendorfer , and G. J. P. Naylor . 2013. “Species Delineation and Global Population Structure of Critically Endangered Sawfishes (Pristidae).” Zoological Journal of the Linnean Society 167: 136–164. 10.1111/j.1096-3642.2012.00872.x.

[ece373733-bib-0015] Fediajevaite, J. , V. Priestley , R. Arnold , and V. Savolainen . 2021. “Meta‐Analysis Shows That Environmental DNA Outperforms Traditional Surveys, but Warrants Better Reporting Standards.” Ecology and Evolution 11: 4803–4815. 10.1002/ece3.7382. 33976849 PMC8093654

[ece373733-bib-0016] Feutry, P. , P. Kyne , R. Pillans , et al. 2015. “Whole Mitogenome Sequencing Refines the Population Structure of the Critically Endangered Sawfish *Pristis pristis* (Linnaeus, 1758).” Marine Ecology Progress Series 533: 237–244. 10.3354/meps11354.

[ece373733-bib-0017] García‐Rodríguez, E. , A. Gonzalez‐Pestana , R. Charles , et al. 2025. “Mapping Important Shark and Ray Areas (ISRAs) in the Central and South American Pacific: Existing Knowledge and Data Needs.” PLoS One 20: e0322445. 10.1371/journal.pone.0322445. 40333947 PMC12058020

[ece373733-bib-0018] Goldberg, C. S. , D. S. Pilliod , R. S. Arkle , and L. P. Waits . 2011. “Molecular Detection of Vertebrates in Stream Water: A Demonstration Using Rocky Mountain Tailed Frogs and Idaho Giant Salamanders.” PLoS One 6: e22746. 10.1371/journal.pone.0022746. 21818382 PMC3144250

[ece373733-bib-0019] Grant, M. I. , P. M. Kyne , C. A. Simpfendorfer , W. T. White , and A. Chin . 2019. “Categorising Use Patterns of Non‐Marine Environments by Elasmobranchs and a Review of Their Extinction Risk.” Reviews in Fish Biology and Fisheries 29: 689–710. 10.1007/s11160-019-09576-w.

[ece373733-bib-0020] Guri, G. , J. L. Ray , A. O. Shelton , et al. 2024. “Quantifying the Detection Sensitivity and Precision of qPCR and ddPCR Mechanisms for eDNA Samples.” Ecology and Evolution 14: e70678. 10.1002/ece3.70678. 39669509 PMC11634988

[ece373733-bib-0021] Instagram . 2022. “Instagram Post by pargos_activos on 07 August 2022.” https://www.instagram.com/reel/Cg8EfrBN40P/?igshid=YmMyMTA2M2Y.

[ece373733-bib-0022] IUCN . 2025. “The IUCN Red List of Threatened Species. Version 2025‐2.” https://www.iucnredlist.org.

[ece373733-bib-0023] IUCN SSC Shark Specialist Group . 2023. Gulf of San Miguel and Tuira River ISRA Factsheet. IUCN SSC Shark Specialist Group. https://sharkrayareas.org/wp‐content/uploads/isra‐factsheets/12CentralSouthPacific/Gulf‐of‐San‐Miguel‐and‐Tuira‐River‐12CentralSouthPacific.pdf.

[ece373733-bib-0024] Jerde, C. L. , A. R. Mahon , W. L. Chadderton , and D. M. Lodge . 2011. ““Sight‐Unseen” Detection of Rare Aquatic Species Using Environmental DNA.” Conservation Letters 4: 150–157. 10.1111/j.1755-263X.2010.00158.x.

[ece373733-bib-0025] Kyne, P. , P. Carlson , R. Aitchison , et al. 2024. “Global Status and Research Priorities for Rhino Rays.” Endangered Species Research 55: 129–140.

[ece373733-bib-0026] Kyne, P. M. , J.‐J. Wang , D. Xiang , X. Chen , and P. Feutry . 2018. “The Phylogenomic Position of the Critically Endangered Largetooth Sawfish *Pristis pristis* (Rhinopristiformes, Pristidae), inferred From the Complete Mitochondrial Genome.” Mitochondrial DNA Part B Resources 3: 970–971. 10.1080/23802359.2018.1501315. 33474383 PMC7799734

[ece373733-bib-0027] Lance, R. F. , and X. Guan . 2020. “Variation in Inhibitor Effects on qPCR Assays and Implications for eDNA Surveys.” Canadian Journal of Fisheries and Aquatic Sciences 77: 23–33. 10.1139/cjfas-2018-0263.

[ece373733-bib-0028] Leeney, R. H. , and P. Poncelet . 2015. “Using Fishers' Ecological Knowledge to Assess the Status and Cultural Importance of Sawfish in Guinea‐Bissau.” Aquatic Conservation: Marine and Freshwater Ecosystems 25: 411–430. 10.1002/aqc.2419.

[ece373733-bib-0029] Lehman, R. N. , G. R. Poulakis , R. M. Scharer , et al. 2022. “Environmental DNA Evidence of the Critically Endangered Smalltooth Sawfish, *Pristis pectinata* , in Historically Occupied US Waters.” Aquatic Conservation: Marine and Freshwater Ecosystems 32: 42–54. 10.1002/aqc.3721.

[ece373733-bib-0030] Lehman, R. N. , G. R. Poulakis , R. M. Scharer , K. E. Schweiss , J. M. Hendon , and N. M. Phillips . 2020. “Environmental DNA Tool for Monitoring the Status of the Critically Endangered Smalltooth Sawfish, *Pristis pectinata* , in the Western Atlantic.” Conservation Genetics Resources 12: 621–629. 10.1007/s12686-020-01149-5.

[ece373733-bib-0031] Lehner, B. , K. Verdin , and A. Jarvis . 2008. “New Global Hydrography Derived From Spaceborne Elevation Data.” Eos, Transactions American Geophysical Union 89: 93–94. 10.1029/2008EO100001.

[ece373733-bib-0032] López‐Angarita, J. , J. Cubillos‐M , M. Villate‐Moreno , et al. 2021. “Bright Spots for Research and Conservation of the Largetooth Sawfish *Pristis pristis* in Colombia and Panamá.” Endangered Species Research 46: 147–160.

[ece373733-bib-0033] López‐Angarita, J. , A. Tilley , J. P. Hawkins , C. Pedraza , and C. M. Roberts . 2018. “Land Use Patterns and Influences of Protected Areas on Mangroves of the Eastern Tropical Pacific.” Biological Conservation 227: 82–91. 10.1016/j.biocon.2018.08.020.

[ece373733-bib-0034] López‐Angarita, J. , M. Villate‐Moreno , J. M. Díaz , J. C. Cubillos‐M , and A. Tilley . 2021. “Identifying Nearshore Nursery Habitats for Sharks and Rays in the Eastern Tropical Pacific From Fishers' Knowledge and Landings.” Ocean and Coastal Management 213: 105825. 10.1016/j.ocecoaman.2021.105825.

[ece373733-bib-0035] Mauvisseau, Q. , J. Davy‐Bowker , M. Bulling , et al. 2019. “Combining ddPCR and Environmental DNA to Improve Detection Capabilities of a Critically Endangered Freshwater Invertebrate.” Scientific Reports 9: 14064. 10.1038/s41598-019-50571-9. 31575968 PMC6773776

[ece373733-bib-0036] Mejía‐Rentería, J. C. , G. A. Castellanos‐Galindo , J. R. Cantera‐Kintz , and S. E. Hamilton . 2018. “A Comparison of Colombian Pacific Mangrove Extent Estimations: Implications for the Conservation of a Unique Neotropical Tidal Forest.” Estuarine, Coastal and Shelf Science 212: 233–240.

[ece373733-bib-0037] Nagarajan, R. P. , M. Bedwell , A. E. Holmes , et al. 2022. “Environmental DNA Methods for Ecological Monitoring and Biodiversity Assessment in Estuaries.” Estuaries and Coasts 45: 2254–2273. 10.1007/s12237-022-01080-y.

[ece373733-bib-0038] Phillips, N. , J. Chaplin , D. Morgan , and S. Peverell . 2009. “Extraction and Amplification of DNA From the Dried Rostra of Sawfishes (Pristidae) for Applications in Conservation Genetics.” Pacific Conservation Biology 15: 128–134. 10.1071/PC090128.

[ece373733-bib-0039] Phillips, N. M. , J. A. Chaplin , D. L. Morgan , and S. C. Peverell . 2011. “Population Genetic Structure and Genetic Diversity of Three Critically Endangered *Pristis* Sawfishes in Australian Waters.” Marine Biology 158: 903–915. 10.1007/s00227-010-1617-z.

[ece373733-bib-0040] Poulakis, G. R. , P. W. Stevens , A. A. Timmers , T. R. Wiley , and C. A. Simpfendorfer . 2011. “Abiotic Affinities and Spatiotemporal Distribution of the Endangered Smalltooth Sawfish, *Pristis pectinata* , in a South‐Western Florida Nursery.” Marine and Freshwater Research 62: 1165–1177. 10.1071/MF11008.

[ece373733-bib-0041] Rački, N. , T. Dreo , I. Gutierrez‐Aguirre , A. Blejec , and M. Ravnikar . 2014. “Reverse Transcriptase Droplet Digital PCR Shows High Resilience to PCR Inhibitors From Plant, Soil and Water Samples.” Plant Methods 10: 42. 25628753 10.1186/s13007-014-0042-6 PMC4307183

[ece373733-bib-0042] Rees, H. C. , B. C. Maddison , D. J. Middleditch , J. R. M. Patmore , and K. C. Gough . 2014. “The Detection of Aquatic Animal Species Using Environmental DNA – A Review of eDNA as a Survey Tool in Ecology.” Journal of Applied Ecology 51: 1450–1459. 10.1111/1365-2664.12306.

[ece373733-bib-0043] Rodrigues, A. É. S. , R. M. S. Brito , P. Charvet , et al. 2025. “Geographical Variation in Mitogenomes of the Largetooth Sawfish *Pristis pristis* : Challenges and Perspectives for Conservation Efforts.” Global Ecology and Conservation 62: e03757. 10.1016/j.gecco.2025.e03757.

[ece373733-bib-0044] Sepulveda, A. J. , P. R. Hutchins , M. Forstchen , M. N. Mckeefry , and A. M. Swigris . 2020. “The Elephant in the Lab (and Field): Contamination in Aquatic Environmental DNA Studies.” Frontiers in Ecology and Evolution 8: 609973. 10.3389/fevo.2020.609973.

[ece373733-bib-0045] Simpfendorfer, C. , P. Kyne , T. Noble , et al. 2016. “Environmental DNA Detects Critically Endangered Largetooth Sawfish in the Wild.” Endangered Species Research 30: 109–116.

[ece373733-bib-0046] Thalinger, B. , K. Deiner , L. R. Harper , et al. 2021. “A Validation Scale to Determine the Readiness of Environmental DNA Assays for Routine Species Monitoring.” Environmental DNA 3: 823–836. 10.1002/edn3.189.

[ece373733-bib-0047] Tingley, R. , R. Coleman , N. Gecse , A. R. van Rooyen , and A. Weeks . 2021. “Accounting for False Positive Detections in Occupancy Studies Based on Environmental DNA: A Case Study of a Threatened Freshwater Fish ( *Galaxiella pusilla* ).” Environmental DNA 3: 388–397. 10.1002/edn3.124.

[ece373733-bib-0048] Valerio‐Vargas, J. , M. Cooper , C. Simpfendorfer , and M. Espinoza . 2025. “Using Environmental DNA to Identify Priority Areas for Sawfish Conservation in Costa Rica, Central America.” Endangered Species Research 57: 161–175.

[ece373733-bib-0049] Wang, Z. , F. Li , F. Wu , et al. 2024. “Environmental DNA and Remote Sensing Datasets Reveal the Spatial Distribution of Aquatic Insects in a Disturbed Subtropical River System.” Journal of Environmental Management 351: 119972. 10.1016/j.jenvman.2023.119972. 38159308

[ece373733-bib-0050] Whitty, J. , J. Keleher , B. Ebner , A. Gleiss , C. Simpfendorfer , and D. Morgan . 2017. “Habitat Use of a Critically Endangered Elasmobranch, the Largetooth Sawfish *Pristis pristis* , in an Intermittently Flowing Riverine Nursery.” Endangered Species Research 34: 211–227. 10.3354/esr00837.

[ece373733-bib-0051] Whitty, J. M. , N. M. Phillips , D. C. Thorburn , et al. 2014. “Utility of Rostra in the Identification of Australian Sawfishes (Chondrichthyes: Pristidae).” Aquatic Conservation: Marine and Freshwater Ecosystems 24: 791–804. 10.1002/aqc.2398.

[ece373733-bib-0052] Wilcox, T. M. , K. S. McKelvey , M. K. Young , et al. 2013. “Robust Detection of Rare Species Using Environmental DNA: The Importance of Primer Specificity.” PLoS One 8: e59520. 10.1371/journal.pone.0059520. 23555689 PMC3608683

[ece373733-bib-0053] Wilcox, T. M. , K. S. McKelvey , M. K. Young , et al. 2016. “Understanding Environmental DNA Detection Probabilities: A Case Study Using a Stream‐Dwelling Char *Salvelinus fontinalis* .” Biological Conservation 194: 209–216. 10.1016/j.biocon.2015.12.023.

[ece373733-bib-0054] Wood, S. A. , X. Pochon , O. Laroche , U. von Ammon , J. Adamson , and A. Zaiko . 2019. “A Comparison of Droplet Digital Polymerase Chain Reaction (PCR), quantitative PCR and Metabarcoding for Species‐Specific Detection in Environmental DNA.” Molecular Ecology Resources 19: 1407–1419. 10.1111/1755-0998.13055. 31293089

[ece373733-bib-0055] Yan, H. F. , P. M. Kyne , R. W. Jabado , et al. 2021. “Overfishing and Habitat Loss Drive Range Contraction of Iconic Marine Fishes to Near Extinction.” Science Advances 7: eabb6026. 10.1126/sciadv.abb6026. 33568471 PMC7875525

[ece373733-bib-0056] Zajac, Z. , B. Stith , A. C. Bowling , C. A. Langtimm , and E. D. Swain . 2015. “Evaluation of Habitat Suitability Index Models by Global Sensitivity and Uncertainty Analyses: A Case Study for Submerged Aquatic Vegetation.” Ecology and Evolution 5: 2503–2517. 10.1002/ece3.1520. 26257866 PMC4523349

[ece373733-bib-0057] Zong, S. , J. Brantschen , X. Zhang , et al. 2024. “Combining Environmental DNA With Remote Sensing Variables to Map Fish Species Distributions Along a Large River.” Remote Sensing in Ecology and Conservation 10: 220–235. 10.1002/rse2.366.

