# Supplementary material for: One Assay to Rule Them All: Development of a Global Environmental DNA Tool to Support Range‐Wide Surveys of an Imperiled Sawfish and Its Application in the Eastern Tropical Pacific
Source: Ecol Evol. 2026 Jun 2;16(6):e73733. doi: 10.1002/ece3.73733 (PMC13239634; doi:10.1002/ece3.73733)
Supplement: Supplementary file 1 — Figure S1: Basin‐mean HSI (blue hue areas) and 95% Confidence Interval (red hue areas) of Habitat Suitability Index (HSI) scores per river basin for all four modeled scenarios for Largetooth Sawfish, Pristis pristis. Scenario 1—Baseline: Only ecological relevant variables. Scenario 2: Kernel Density Estimation (KDE) 10% variable weight in the HSI‐weighted geometric mean (WGM) model. Scenario 3: KDE 20%. Scenario 4: KDE 40%. Figure S2: Habitat suitability classification for all scenarios. River basins were classified into High, Moderate, or Low suitability for Largetooth Sawfish, Pristis pristis , based on the mean Habitat Suitability Index (HSI) and the probability of exceeding a global threshold calculated from Monte Carlo simulations. (A) Scenario 1—Baseline: Only ecologically relevant variables. (B) Scenario 2: Kernel Density Estimation (KDE) 10% variable weight in the HSI‐weighted geometric mean (WGM) model. (C) Scenario 3: KDE 20%. (D) Scenario 4: KDE 40%. [file ECE3-16-e73733-s002.pdf]

Cubillos-M., J. C., Fearing, A., Chapman, D. D., Kyne, P. M., Dias, P. J., Gonzalez, C., Lehman, R. N., Chichaco, Y., Huerta-Beltrán, B. L., McCulloch, K. L., Toepfer, S. M., Carlson, J. K., Phillips, N. M. (2026).One assay to rule them all: development of a global environmental DNA tool to support range-wide surveys of an imperiled sawfish and its application in the Eastern Tropical Pacific. *Ecology and Evolution*

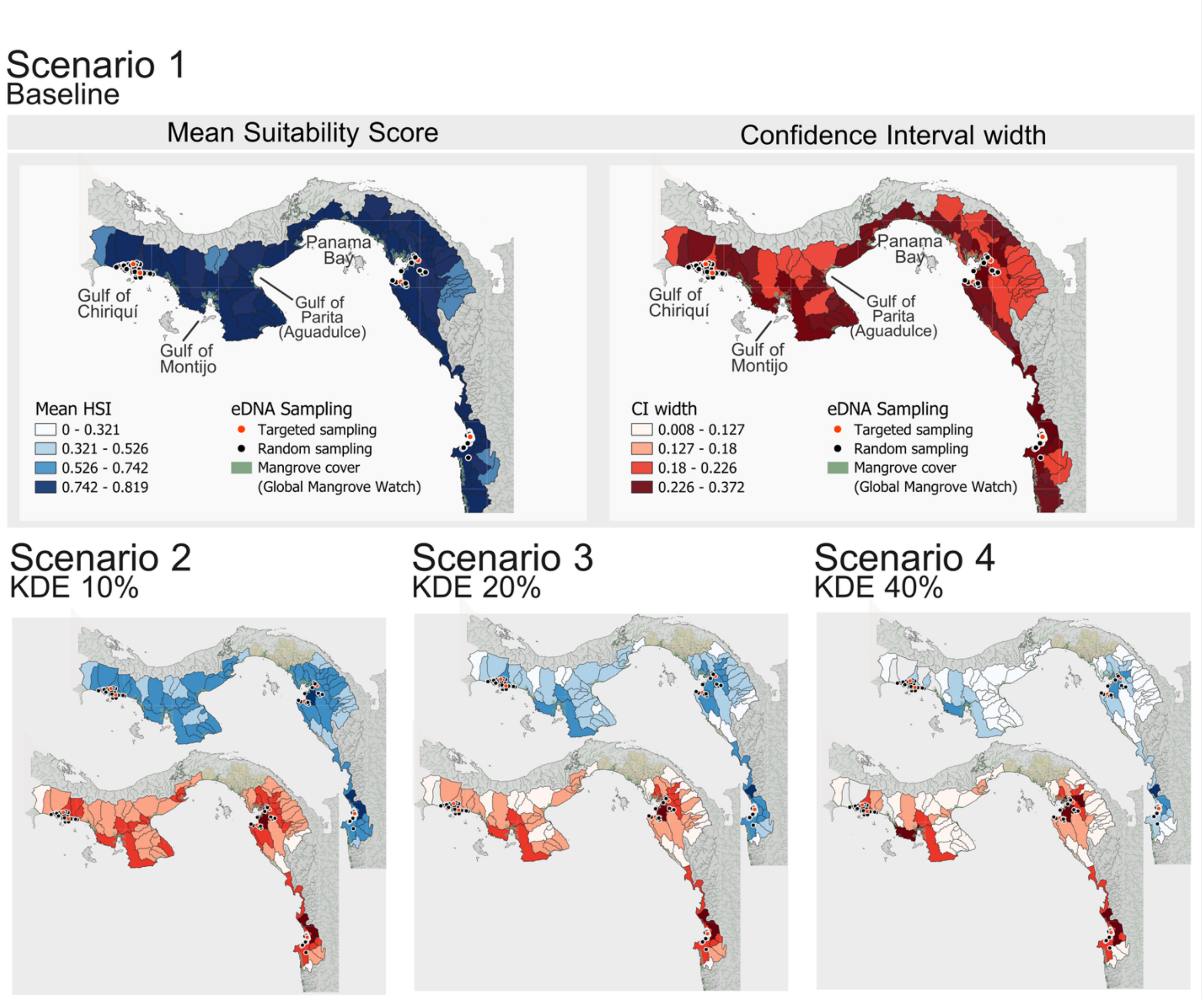


**Figure S1.** Basin-mean HSI (blue hue areas) and 95% Confidence Interval (red hue areas) of Habitat Suitability Index (HSI) scores per river basin for all four modelled scenarios for Largetooth Sawfish, *Pristis pristis.* Scenario 1 - Baseline: Only ecological relevant variables. Scenario 2: Kernel Density Estimation (KDE) 10% variable weight in the HSI-weighted geometric mean (WGM) model. Scenario 3: KDE 20%. Scenario 4: KDE 40%.

Cubillos-M., J. C., Fearing, A., Chapman, D. D., Kyne, P. M., Dias, P. J., Gonzalez, C., Lehman, R. N., Chichaco, Y., Huerta-Beltrán, B. L., McCulloch, K. L., Toepfer, S. M., Carlson, J. K., Phillips, N. M. (2026).One assay to rule them all: development of a global environmental DNA tool to support range-wide surveys of an imperiled sawfish and its application in the Eastern Tropical Pacific. *Ecology and Evolution*

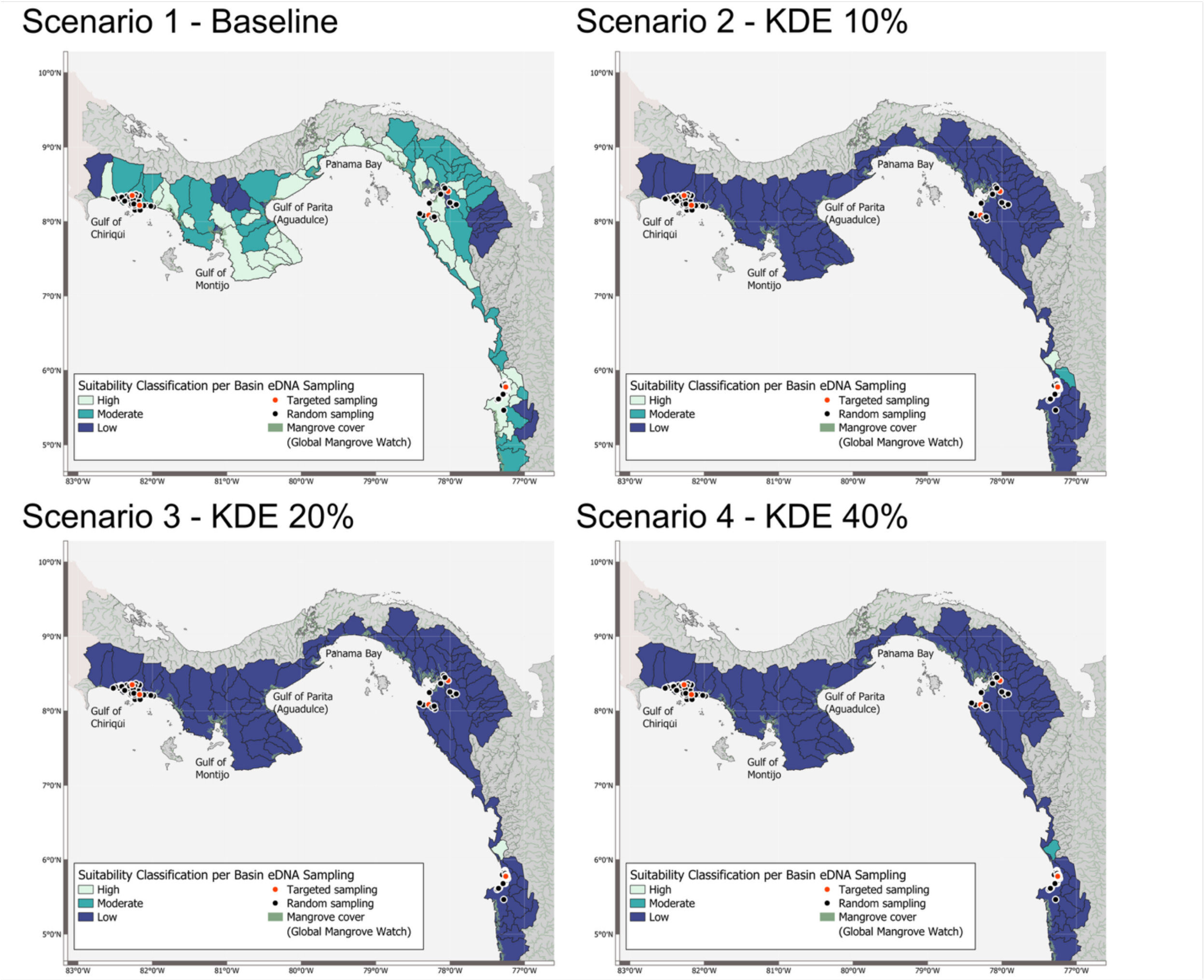


**Figure S2.** Habitat suitability classification for all scenarios. River basins were classified into *High*, *Moderate*, or *Low* suitability for Largetooth Sawfish, *Pristis pristis*, based on the mean Habitat Suitability Index (HSI) and the probability of exceeding a global threshold calculated from Monte Carlo simulations. A) Scenario 1 - Baseline: Only ecologically relevant variables. B) Scenario 2: Kernel Density Estimation (KDE) 10% variable weight in the HSI-weighted geometric mean (WGM) model. C) Scenario 3: KDE 20%. D) Scenario 4: KDE 40%.
